# Curcumin in Liver Diseases: A Systematic Review of the Cellular Mechanisms of Oxidative Stress and Clinical Perspective

**DOI:** 10.3390/nu10070855

**Published:** 2018-07-01

**Authors:** Mohammad Hosein Farzaei, Mahdi Zobeiri, Fatemeh Parvizi, Fardous F. El-Senduny, Ilias Marmouzi, Ericsson Coy-Barrera, Rozita Naseri, Seyed Mohammad Nabavi, Roja Rahimi, Mohammad Abdollahi

**Affiliations:** 1 Pharmaceutical Sciences Research Center, Kermanshah University of Medical Sciences, Kermanshah 6734667149, Iran; parvizi_70@yahoo.com; 2 Internal Medicine Department, Imam Reza Hospital, Kermanshah University of Medical Sciences, Kermanshah 6734667149, Iran; mehdizobeiri@yahoo.com (M.Z.); rnasseri.75@gmail.com (R.N.); 3 Biochemistry division, Chemistry Department, Faculty of Science, Mansoura University, Mansoura 35516, Egypt; biobotany@gmail.com; 4 Laboratory of Pharmacology and Toxicology Faculty of Medicine and Pharmacy, Mohammed V University in Rabat, Rabat 10100, Morocco; ilias.marmouzi@gmail.com; 5 Bioorganic Chemistry Laboratory, Facultad de Ciencias Básicas y Aplicadas, Universidad Militar Nueva Granada, Campus Nueva Granada, Cajicá 250247, Colombia; ericsson.coy@unimilitar.edu.co; 6 Applied Biotechnology Research Center, Baghyatollah University of Medical Sciences, Tehran 1435916471, Iran; Nabavi208@gmail.com; 7 Department of Persian Pharmacy, School of Traditional Medicine, Tehran University of Medical Sciences, Tehran 1416663361, Iran; rojarahimi@gmail.com; 8 Toxicology and Diseases Group, The Institute of Pharmaceutical Sciences (TIPS) and Department of Toxicology and Pharmacology, Faculty of Pharmacy, Tehran University of Medical Sciences, Tehran 1417614411, Iran

**Keywords:** curcumin, hepatotoxicity, liver diseases, oxidative stress, systematic review

## Abstract

Oxidative stress has been considered a key causing factor of liver damage induced by a variety of agents, including alcohol, drugs, viral infections, environmental pollutants and dietary components, which in turn results in progression of liver injury, non-alcoholic steatohepatitis, non-alcoholic liver disease, liver fibrosis and cirrhosis. During the past 30 years and even after the major progress in the liver disease management, millions of people worldwide still suffer from an acute or chronic liver condition. Curcumin is one of the most commonly used indigenous molecules endowed by various shielding functionalities that protects the liver. The aim of the present study is to comprehensively review pharmacological effects and molecular mechanisms, as well as clinical evidence, of curcumin as a lead compound in the prevention and treatment of oxidative associated liver diseases. For this purpose, electronic databases including “Scopus,” “PubMed,” “Science Direct” and “Cochrane library” were extensively searched with the keywords “curcumin or curcuminoids” and “hepatoprotective or hepatotoxicity or liver” along with “oxidative or oxidant.” Results showed that curcumin exerts remarkable protective and therapeutic effects of oxidative associated liver diseases through various cellular and molecular mechanisms. Those mechanisms include suppressing the proinflammatory cytokines, lipid perodixation products, PI3K/Akt and hepatic stellate cells activation, as well as ameliorating cellular responses to oxidative stress such as the expression of Nrf2, SOD, CAT, GSH, GPx and GR. Taking together, curcumin itself acts as a free radical scavenger over the activity of different kinds of ROS via its phenolic, β-diketone and methoxy group. Further clinical studies are still needed in order to recognize the structure-activity relationships and molecular mechanisms of curcumin in oxidative associated liver diseases.

## 1. Introduction

During the past 30 years and even after the major progress in the liver disease management, millions of people still suffer from an acute or chronic liver condition worldwide. Liver diseases affect more than 10% of the world population and its mortal end-stage generally follows cirrhosis and liver cancer [1]. Diverse etiologies characterize the disease to constitute about the fourth to the fifth cause of deaths worldwide. The Nonalcoholic Fatty Liver Disease (NAFLD) is the global leading cause of liver diseases with 40% frequently, followed by Hepatitis B virus (HBV), Hepatitis C virus (HCV) and harmful alcohol consumption, accounting for 30%, 15% and 11%, respectively [1].

Chronic liver diseases are often accompanied by increased oxidative stress, irrespective of the cause of the liver dysfunction [2]. Oxidative stress (indicating excessive reactive oxygen species (ROS) levels and an oxidant and antioxidant imbalance) can lead to cellular degradation of proteins, lipids and DNA. Reactive oxygen species (ROS) participates in the liver fibrogenic response and contributes to ischemia/regeneration, necrosis and apoptosis. These modifications result in altered gene expression and progressive liver damage [2].

Natural products provide a repertory for discovery of new leads that can be used in treating different types of diseases such as cancer, inflammation and liver diseases. More than half of all pharmaceutical products have been discovered from natural compounds or their derivatives [3]. In the United States and Europe, approximately 65% of patients use herbal medicines against liver disease, due to their wide availability, low toxicity, pharmacological activity and chemical diversity and low side effects compared to synthetic drugs [4,5,6,7,8]. Curcumin is the main constituent of turmeric, the rhizome of *Curcuma longa*. It is widely used due to its therapeutic effectiveness and acceptable safety specification [9,10]. Curcumin possesses several biological activities such as anti-inflammatory [11,12], anticancer [13], antioxidant [14] and the ability to heal wounds [15]. From these facts, the aim of this review is to compile and discuss the effects of curcumin for the prevention and treatment of oxidative associated liver diseases as well as to highlight its molecular mechanism of action.

## 2. Liver Disease: Pathophysiology and Epidemiology

The vitality of the human liver is mainly associated with the impressive processes attributed to this part of the entrails. Its nomination was even regarded as a synonym of life [1]. In fact, multiple functionalities are attributed to this triangular organ extending across the abdominal cavity below the diaphragm [1]. Its metabolic and secretory capacities can cause hepatocellular death and eventually liver disease by involving a prominent exposure to alcohol, dietary components and viral infections [16]. Apparently, this organ is extremely vulnerable to numerous pathologies mainly associated with its great number of functions, structural organization, strategic localization and cell sensitivities. A number of mechanisms such as direct damage, stimulation of immune response against cells, formation of reactive intermediates, cytoskeletal damage, disruption of normal cell metabolism, triggering of apoptosis and hypoxia are involved in hepatocellular injury.

Hepatitis is an inflammatory process caused by drugs, alcohol and often by a virus. This is commonly known as hepatitis A, B, C, D and E. For instance, it is estimated that between 130 and 150 million patients suffer from HCV infection globally [1]. Chronic hepatitis therefore occurs when the injury persists for more than 6 months, resulting in raising aminotransferase levels or viral markers [17]. On the other hand, the abnormal accumulation of lipids in hepatic cells (5% or more) is generally referred as steatosis. The macrovesicular steatosis generates the propitious environment of alcoholic and non-alcoholic steatohepatitis (ASH & NASH) lesion development [18,19]. NASH is associated with hepatic steatosis and inflammation [17], however ASH and NASH are hardly distinguished on single-handed histological grounds [20]. Their pathophysiology includes hepatocellular damage most severe in (or restricted to) perivenular areas, inflammation and fibrosis. In this sense, the nomenclatures alcoholic fatty liver disease (AFLD) and NAFLD are used to demonstrate a wide range of alterations between uncomplicated steatosis and cirrhosis [21]. NAFLD is a chronic liver disease in non-abusively alcohol-drinking patients. It is highly associated with obesity, type-II diabetes mellitus and hyperlipidemia (high levels of triglycerides, low-density lipoprotein cholesterol, cholesterol and low level of high-density lipoprotein cholesterol) [22]. NAFLD treatment mainly depends on the severity. Regardless whether it is treated or not, NAFLD may progress to fibrosis and cirrhosis in specific population and the risk increases with aging, diabetes and obesity [23,24,25]. Current treatments rely on increasing insulin sensitivity (thiazolidinediones and metformin), slow-rate lowering body weight, reducing lipid level (gemfibrozil) and protecting hepatocytes (ursodeoxycholic acid, betaine, *N*-acetylcysteine, vitamin E). Thiazolidinediones (TZD) increases insulin sensitivity and lipid oxidation; preventing their accumulation in the liver [26,27]. Unfortunately, TZD has adverse side effects leading to edema and increases the body weight [26,28]. Therefore, there is a need for new strategies to control those liver diseases such as NAFLD, cirrhosis and even liver cancer [29,30].

## 3. Role of Oxidative Stress in Development of Liver Disease

The pathogenesis of liver diseases integrates the oxidative stress processes and damages, including lipids, proteins and DNA alterations as well as modulation of functional signaling pathways. Oxidative stress is an imbalance between antioxidant capacity and the level of ROS in cells. ROS and reactive nitrogen species (RNS) are now being used to describe the free radicals derived from molecular oxygen and the oxidants derived from NO•, respectively [31,32]. Additionally, ROS can be produced by the endoplasmic reticulum in the liver via the cytochrome P450 enzymes at the macrophages and neutrophils levels [2]. One of the mechanisms leading to oxidative stress is the modulation of protein expression under H_2_O_2_ stress. This alteration exposes the liver to severe oxidative stress, resulting in hepatocyte apoptosis. Oxidative stress induced liver diseases can also cause brain impairment and kidney failure [33,34].

Many etiological factors were associated with liver disease and are highly productive of ROS. In fact, studies have demonstrated that ethanol can result in a significant rising of mitochondrial ROS levels in hepatocytes [35]. Ethanol oxidation involves at least three distinct enzymatic pathways [36]. Alcohol dehydrogenase (ADH) firstly oxidizes ethanol to acetaldehyde and the latter is then oxidized to acetate by aldehyde dehydrogenases (ALDH) in the mitochondria. The ethanol oxidation is also promoted by the microsomal ethanol oxidizing system (MEOS), the cytochrome P450 enzyme Cytochrome P450 2E1 (CYP2E1) and the catalase in peroxisomes. An excessive consumption of alcohol results in a higher metabolic activity which elevates ROS and liver injury [37].

On the other hand, drug hepatotoxicity is linked to ROS and RNS productions [38]. Actually, the intake of medication can induce oxidative stress via increase of cellular oxidants and lipid peroxidation and depletion of antioxidants in the liver. For instance, sulfasalazine reduces superoxide dismutase (SOD) but increases catalase (CAT) activity. Zoledronic acid results in significantly elevated malondialdehyde (MDA) and nitric oxide levels, whereas reduced glutathione (GSH) levels [39]. Even paracetamol also increases MDA, nitrite and nitrate in the liver and reduces total SOD and Cu/Zn-SOD activities. In addition to these factors, exposure to heavy metals, microcystin, radiation and temperature have also been shown to cause oxidative damage in liver [40,41].

## 4. Curcumin and Oxidative Stress

*Curcuma longa* (turmeric) is a widely used spice, coloring agent and source of curcumin [42]. Derivatives from *Curcuma longa* L. are including, Curcumin, Ar-turmerone, Methylcurcumin, Demethoxy curcumin, Bisdemethoxy curcumin and Sodium curcuminate. Curcumin that is in the most important fraction of *Curcuma longa* L., is one of the most commonly used indigenous molecules endowed by various protective functionalities [42,43]. The pharmacokinetic (PK) studies of curcumin have consistently reported that the bioavailability of curcumin is relatively low due to its instability, poor solubility and absorption and its rapid metabolic elimination by reduction and conjugation in the presence of mild temperature and light. Similar to rodent studies, the poor bioavailability of curcumin in humans causes a primary barrier to achieve adequate plasma levels with favorable pharmacological effects [42,44]. Hence curcumin derivatives are of great interest in biomedical research [43].

This bright-yellow curcuminoid contains a variety of functional antioxidant groups, including the β-diketo group, carbon-carbon double bonds and phenyl rings. Curcumin can thus eliminate lipid radicals in the cell membrane and become a phenoxyl radical, so it is considered a very strong lipid-soluble antioxidant [45]. Furthermore, curcumin was found to inhibit lipid peroxidation and neutralize ROS (superoxide, peroxyl, hydroxyl radicals) [46] and RNS (nitric oxide and peroxynitrite) [47]. The protective effect of curcumin against oxidative stress was previously described in vitro and in vivo [48,49,50,51]. For instance, its biomembrane-protective effect against peroxidative damage was mainly linked to its ROS scavenging ability [52]. The hydrogen donor capacity was also associated with its phenolic and/or central methylenic groups. The enol form of curcumin was demonstrated to be significantly more stable than the diketo form. This study also suggested that the hydrogen atom abstraction takes place in the phenolic group [52,53,54,55]. However, the activity of the radical and the reaction medium influenced the relative contribution of the phenolic group and the central methylenic group to the antioxidant activity [52,56]. In addition, curcumin degradation products can also claim for its antioxidant activities. It can degrade under basic pH after 30 min into ferulic acid and vanillin [57]. In addition, curcumin exhibited chelating activity and is able to capture ferrous ion through its functional carbonyl groups [45].

## 5. Study Design

**Figure 1:**
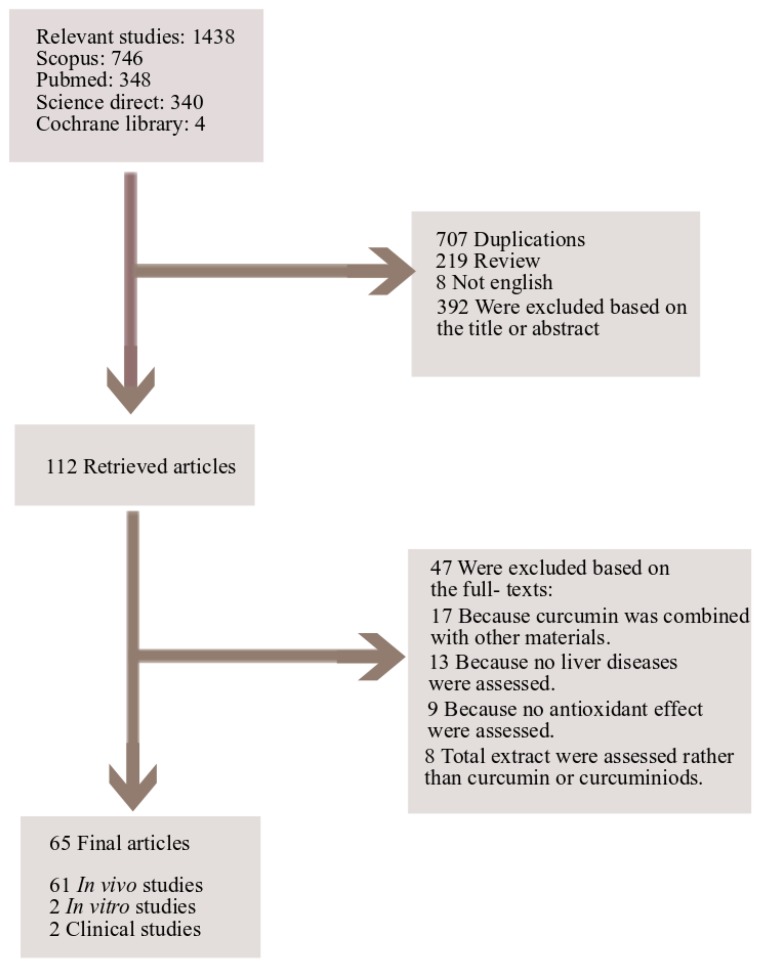
Study selection diagram.

Electronic databases including “Scopus,” “PubMed,” “Science Direct” and “Cochrane library” were searched for cellular, animal or human studies with the keywords “curcumin or curcuminoids” and “hepatoprotective or hepatotoxicity or liver” in title/abstract, along with “oxidative or oxidant” in the whole text. References of the final articles were also reviewed for more relevant articles. Data were collected from the inception date until 2018 (up to January). Only English language papers were included. Results from primary systematic search were screened by two independent investigators. All published articles as well as abstracts presented at meetings were evaluated. From a total of 1436 results, 707 were excluded because of duplication, 219 for being reviews and 392 being irrelevant judged on the title and/or abstract. From the 112 primarily selected papers, 47 were excluded based on the full texts. Finally, 65 articles were included in this review. Figure 1 illustrates a flow diagram of study selection process.

## 6. Cellular and Molecular Mechanisms of Curcumin in the Prevention of Oxidative Associated Liver Disease

### 6.1. Curcumin and Non-Alcoholic Steatohepatitis (NASH)

The relationship between steatosis and fibrosis, hepatic cell injury and lobular inflammation is recognized as NASH. On this context, curcumin (200 mg/kg/day for 3 weeks) likewise exerted a protective effect on CCl_4_-induced NASH. During the respective histopathological inspection, depletions of lipid accumulation and MDA deposition were observed in male Wistar-Albino rats [58]. Curcumin also restricted successfully the fibrosis (both development and progression) in mice with methionine+choline-deficiency (MCD)-induced steatohepatitis [59]. Reductions of tissue inhibitor metalloproteinase-1 (TIMP-1) secretion and inhibition of 8-OH-deoxyguanosine-mediated hepatic oxidative stress in cultured stellate cells from mice were recorded [59]. In this regard, the hepatic inflammation and steatosis was then decreased along with the diminished levels of serum biochemical markers and cytokines and enlarged levels of liver antioxidants [60]. Furthermore, the nuclear factor erythroid 2 related factor 2 (Nrf2) protein expression was significantly higher in curcumin-treated rats, so authors concluded this NASH prevention/amelioration can be related with Nrf2 activation [60] as described for heavy metals-induced hepatotoxicity. A study was designed to investigate the underlying mechanisms of curcumin-treating protection on NASH progression using an innovative NASH-hepatocellular carcinoma (HCC) mouse model [61]. As result of this model, curcumin reduced the steatosis and fibrosis conditions and, as previously mentioned in several studies, caused a prominent decrease of biomarkers in NASH mice. However, the most important finding of this study was that curcumin can modulate/inhibit the high-mobility group box 1 (HMGB1)-NF-κB translocation to prevent the NASH progression and liver injury [61].

### 6.2. Curcumin and Alcoholic Liver Disease (ALD)

Morphological changes and clinical disorders in liver can be produced by the metabolism-related toxic effects of ethanol, since its oxidation affords apoptosis and cell injury-initiating products (e.g., acetaldehyde and ROS). These products can lead to fatty liver, hepatic inflammation, alcoholic hepatitis (necrosis) and progressive alcoholic cirrhosis (fibrosis). Aforementioned facts comprise the well-known alcoholic liver disease (ALD) [62]. Curcumin has been studied in several investigations for attenuating ALD effects and describing the respective mechanisms. Thus, inflammation and liver pathology can be improved in curcumin-administered (400 or 1200 mg/kg/day orally for 4 weeks) female Sprague-Dawley rats. Decreasing of hepatic MDA levels and inhibition of NF-κB activation were also observed for both doses but lower ones could avoid hepatocyte apoptosis [63]. Curcumin additionally modulated the antioxidant capacity, alcohol metabolic enzyme activity (i.e., CYP2E1 inhibition) and lipid metabolism (i.e., increasing activated protein kinase (AMPK) expression) after chronic alcohol intake-induced liver injury [64]. Curcumin was also related to hepatoprotection acting as a redox regulator and time/dose-dependent heme oxygenase-1 (HO-1) inducer against ethanol-induced oxidative injury in hepatocytes [65]. In balb/c mice, curcumin has been also suggested to defend liver from chronic-ethanol-induced damage. It also mitigated hepatohistopathological changes and lipid accumulation and amended levels of common biomarkers [66]. The attenuation of liver oxidative stress by curcumin has been described via induction/modulation of antioxidant signaling pathways such as Nrf2 activation and up-regulation of detoxifying genes expression (e.g., NQO1, HO-1 and GCLC) via ERK/p38-MAPK pathways [67]. Moreover, curcumin treatment (150 mg/kg/day for 8 weeks) exhibited health benefits in female Wistar-Furth rats through lipid metabolism modulation. Curcumin treatment alleviated the hepatosteatosis and suppressed the atherogenesis in ALD, even enhancing the antiatherogenic markers, that is, PON1/HTLase and GSH [68].

However, despite the potential of curcumin as an anti-inflammatory and antifibrotic agent against liver damage, some concentration-dependent negative effects were reported in male C57BL/6 mice. Based on those findings, curcumin seems to have dual impacts on alcoholic liver injury. The recognized hepatoprotective effect of curcumin was achieved at 0.1 mM, whereas an acceleration of liver injury and cellular edema was observed using 1 mM dose [69]. These facts can therefore be considered as a caution on using high doses of traditional medicines. In this sense, literature has a plethora of positive effects for more affordable therapeutics but negative ones are infrequently reported.

### 6.3. Curcumin for the Prevention of the Oxidative Stress in Liver

**Figure 2:**
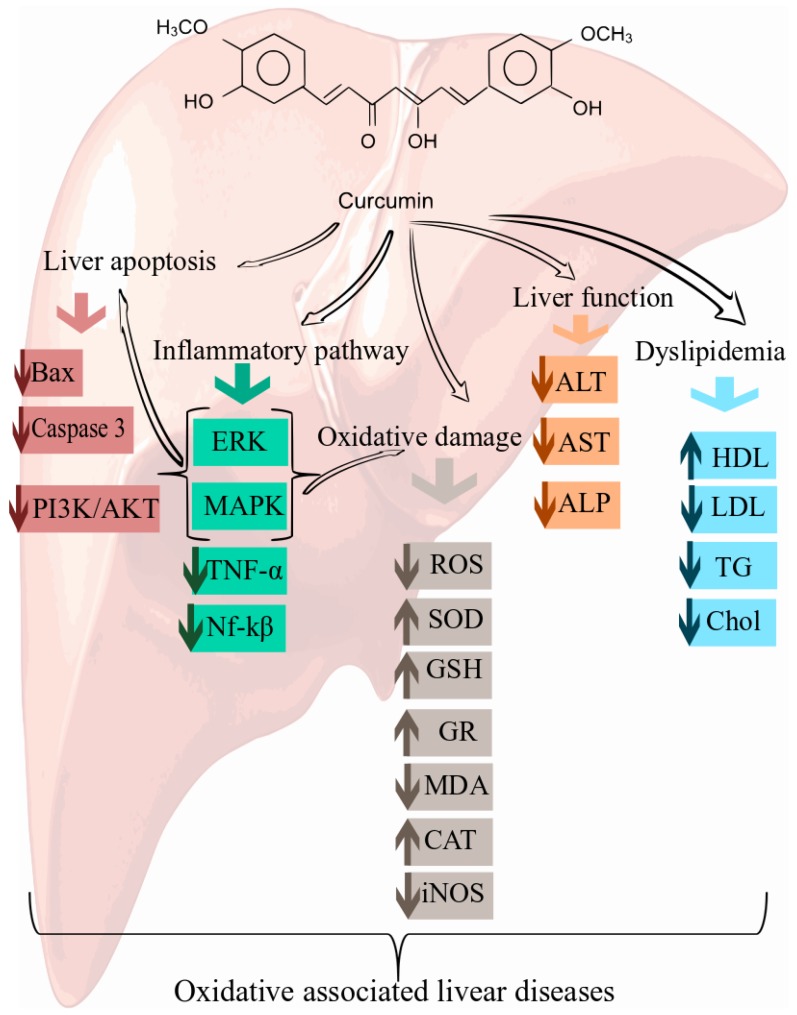
Cellular and molecular mechanisms of curcumin in the prevention of oxidative-associated liver disease. Alanine aminotransferase (ALT), aspartate aminotransferase (AST), alkaline phosphatase (ALP), reactive oxygen species (ROS), sulfasalazine reduces superoxide dismutase (SOD), glutathione (GSH), glutathione reductase (GR), malondialdehyde (MDA), catalase (CAT), inducible nitric oxide (iNOS), high-density lipoprotein (HDL), low-density lipoprotein (LDL), triglyceride (TG), extracellular signal-regulated kinases (ERK), mitogen-activated protein kinase (MAPK).

The imbalance between the generation and degradation of ROS can cause oxidative stress and eventually the generation of free radicals and cellular damage. However, as will be explained later, it has been reported that curcumin can be used for the prevention of oxidative stress in the liver. Some of the important mechanisms of curcumin in the prevention of oxidative associated liver disease are shown in Figure 2. Hydrogen peroxide was used by AL-Rubaei and colleagues as an agent that can damage liver cells. Their result showed that curcumin prevented liver toxicity and lower levels of alanine aminotransferase (ALT), aspartate aminotransferase (AST) and alkaline phosphatase (ALP) enzymes could be measured [70]. The effect of the pre- and post-treatment of curcumin were examined in rats contacted with methotrexate (MTX) induced oxidative stress. The results showed that the amelioration of antioxidant enzymes, including glutathione *S*-transferase (GST), glutathione reductase (GR), glutathione peroxidase (GPx), SOD and CAT, also the inhibition of ROS production can prevent oxidative stress in pre-treatment. Curcumin post-treatment can control the balance between oxidant and antioxidant [71]. In another work, the ability of curcumin was shown to treat hepatotoxicity caused by methotrexate [72]. AL-Harbi et al. found that administration of curcumin (60 mg/kg) have hepatoprotective effects on sodium fluoride induced oxidative stress. It can reduce hepatotoxicity and liver enzyme activities [73]. The effect of curcumin against oxidative stress induced by malathion (MAL), an organophosphorus insecticide (OPI), has been reported to reduce the MDA and nitric oxide (NO) levels and increased the GSH levels [74]. Excessive accumulation of iron overload (IOL) in the liver leads to oxidative stress as a result of cellular injury. The result of a study performed by Ali Hussein and colleagues indicated that treatment of rats with oxidative damage induced by IOL, significantly increased serum unsaturated iron binding capacity (UIBC), total protein, albumin, GSH, SOD and CAT activity accompanied by a reduction in serum iron, total iron-binding capacity (TIBC), transferrin (TF), ALT and AST activities, NO and MDA levels [75]. Similarly, in another study, the hepatoprotective effect of curcumin against oxidative stress created by IOL was confirmed [76]. Ciftci et al. in a rat model of oxidative stress induced by 2,3,7,8-tetrachlorodibenzo-p-dioxin (TCDD), a persistent environmental pollutant, noticed that the administration of 100 mg/kg/day curcumin reduced liver level of thiobarbituric acid reactive substances (TBARS). TCDD also increased liver levels of GSH, CuZn-SOD and GSH-Px [77]. Coneac et al. found that curcumin is able to reduce oxidative stress in acute experimental inflammation produced by Turpentine oil [78]. Dai et al. has confirmed that curcumin could ameliorate reduced L02 cell viability, prevent oxidative stress and inhibit the rises of SOD activity and GSH level. It could also be used as a prospective medical factor for quinocetone (QCT)-induced oxidative stress in human hepatocyte L02 cells [79]. In another in vitro study, the ability of curcumin to suppress oxidative stress in rat hepatic stellate cells (HSCs)-T6 was tested. Secretion of extracellular matrix (ECM) molecules and the levels of ROS and MDA were found to be decreased, whereas nuclear expression levels of Nrf2 and the levels of GSH were increased. Additionally, the expression of smooth muscle α-actin (α-SMA) was suppressed [80]. It has been demonstrated that curcumin possesses the ability to attenuate lipid peroxidation and increase GSH level in rats exposed to cadmium, an industrial pollutant [81]. An increase of GSH level and SOD, GPx, GR and CAT activities and decrease of MDA level were also observed with curcumin in an immobilization-induced stress rat model, which changes the activities of antioxidant enzymes [82]. In carbon tetrachloride (CCl_4_)-induced liver injury and fibrogenesis model, it was demonstrated that curcumin could be effective in protecting the liver by reducing oxidative stress. Activity of NF-κB, production of proinflammatory cytokines, structure of liver tissue and activation of HSC were then inhibited [83,84]. Singh et al. indicated that curcumin can be a potent protective agent against lindane as a pesticide that induces hepatotoxicity in rats. The presence of curcumin (pre- and post-treatment) with lindane significantly normalized the increased lipid peroxidation and decreased CAT, GPx, GR and SOD activities [85]. The possible protective role of curcumin was evaluated on cypermethrin-induced oxidative damage. A significant decrease in lipid peroxidation and the blood biochemical markers and decrease in CAT, GPx and GSH levels was observed in the liver of rats exposed to cypermethrin [86]. Watanab et al. determined the possibilities of curcumin in preventing or minimizing the oxidative stress induced by administration of trichloroethylene (TCE) in mouse liver. The activities of antioxidative enzymes were thus measured after curcumin protection [87].

### 6.4. Curcumin and Liver Injury

Curcumin and related phenolics have been associated with the inhibition of lipid peroxidation, free radical formation and DNA damage under the role of radical scavengers and/or antioxidants. However, additional examination has been suggested to be explored in order to elucidate the mechanisms for the beneficial properties of curcumin on several oxidative stress-associated diseases such as liver injury. Thus, protective effects of curcumin on liver injury induced by different factors have been described by several studies using murine models. Association with the HO-1, an important mediator of cytoprotective events, might be considered as a plausible explanation about the way curcumin can act. Hepatoprotection of curcumin (single dose at 100 mg/kg intra peritoneal) against lipopolysaccharide-induced hepatitis in d-galactosamine-sensitized rats was clearly connected with the HO-1 up-regulation and the consequently bilirubin production. This protective effect accordingly impacted on nitric oxide synthase 2 (NOS-2) down-regulation and reduced the amounts of NO and lipid peroxidation products in liver [88].

Relatedly, prophylactic administration of curcumin to mice (300 mg/kg/day orally for 7 days) resulted in a reasonable chemopreventive capacity for protection of microcystins-induced liver injury [89]. The statistically significant reduction of ALT, lactate dehydrogenase (LDH) and GST levels in comparison to that of a microcystins-treated group were additionally observed. Positive MDA and negative SOD variations were also detected, so the authors concluded that curcumin can improve the hepatic antioxidative abilities in mice [89]. Comparable kind of reduction in oxidative stress and DNA damage was furthermore observed in Wistar albino rats (50 mg/kg/day orally for 14 days), since the biomarkers levels after biliary blocking were diminished in curcumin-treated rats. This result was a clear indication that curcumin assisted the recovery of liver function parameters, specifically on protecting the cholestasis-caused damages. In addition, curcumin generated a further reduction of ductal proliferation and portal inflammation in comparison with the induced group after histopathological evaluation. These facts indicated correspondingly a valuable decreasing of the inflammatory process. Similarly, after biliary duct ligation (BDL)-induced liver injury, curcumin promoted the statistically significant reduction of MDA, glutathione, NO and tumor necrosis factor-α (TNF-α) levels and enhancing the catalase, SOD and GST activities in liver [90]. Another study in liver tissue of Wistar rats also evidenced that curcumin can exert protection on BDL-induced hepatic damage through antioxidant action [91]. The down-regulation of Rho-related C3 botulinum toxin substrate (Rac1), NADPH oxidase-1 (NOX1) and Rac1-guanosine triphosphate (Rac1-GTP) and enhanced levels of hepatic enzymes (i.e., ALP, AST, ALT) and antioxidants (i.e., thiols, SOD and catalase) were also identified [91]. Additionally, hepatoprotective effect against ischemia/reperfusion (I/R)-promoted liver injury was found upon curcumin pretreatment (100 mg/kg IP 30 min prior to I/R) in Female Wistar Albino rat. Thus, the reduction of some liver injury indexes in blood (i.e., NO, TNF-α, methyl guabidine (MG), glutamic oxaloacetic transaminase (GOT), glutamate pyruvate transaminase (GPT) and LDH) were also described as an indication of the potential of curcumin against inflammatory responses and oxidative/nitrosative stress during reperfusion liver injury [92].

Discrepancy between ineffective antioxidant defense and enlarged free radical production in liver has been evidently linked with drugs exposure-related oxidative stress [93]. In this sense, curcumin (three doses at 200 mg/kg during 36 h) also triggered attenuated levels of biochemical parameters in liver damage induced by acetaminophen overdose toxicity (at 750 mg/kg orally) in albino western rats. Particularly, down-regulation of pro-apoptotic Bax and macrosialin cluster of differentiation 68 (CD68) protein expressions were observed through immunohistochemical evaluations [94]. The hepatoprotective results were increased whether curcumin was combined with thymoquinone, comprising fewer side effects in comparison to *N*-acetylcysteine (i.e., the best antidote for acetaminophen hepatotoxicity). Similar results were found on gentamicin-induced liver injury in male albino rats, since curcumin administration (20 mg/kg/every other day, oral for 21 days) exhibited a statistically significant reduction of levels of biochemical parameters, TNF-α and bilirubin. Liver histological alterations and apoptotic executioner caspase-3, the proapopototic Bax and antiapoptotic B-cell lymphoma-2 (Bcl-2) protein expressions were successfully amended in curcumin-treated animals [95].

Several reports have also recognized the protective capacity of curcumin on liver injury produced by some xenobiotics. Curcumin (100 or 200 mg/kg/day intra peritoneal) has been previously described to exhibit a significant hepatoprotective activity against liver damages induced by aflatoxin B1 [96], lambda cyhalothrin [97], CCl_4_ [98,99], mercury [100] and other heavy metals [101] in adult rats. Levels of lipid peroxidation, serum biomarker enzymes, liver MDA, hydroxyproline and liver antioxidants (i.e., GSH, SOD, catalase) were correspondingly modified after curcumin treatment. DNA fragmentation preventing and mitochondrial functionalities preserving were also explained according to those findings.

Regarding protective ability of curcumin against heavy-metals (such as copper, cadmium, chromium, mercury, lead and arsenic), its free radical scavenging/reducing power/chelating capacities can restrain the heavy-metals-promoted hepatotoxicity by liver oxidative stress-related antagonism. These abilities were connected with the induction of the Nrf2/antioxidant response elements/Kelch-like ECH associated protein 1 (Nrf2/ARE/Keap1) pathway [100,101]. On the other hand, curcumin also exhibited a dose-dependent protection in CCl_4_-induced liver damage of Jian carp (*Cyprinus carpio* var. Jian). The up-regulation of the common antioxidative activities (SOD and GSH) and inhibition of cytokine production (e.g., Interleukin-1β (IL-1β), TNF-α, Interleukin 12 (IL-12)) were then observed and consequently correlated to the exhibited protective effect [102].

Despite the enormous, evidenced potential of curcumin to impede liver injury, the investigations are still quite scarce. The related studies are limited to evaluate the blood and liver levels of some biomarkers and examine liver histopathological/immunohistochemical results. Further studies on molecular, cellular and physiological mechanisms during curcumin hepatoprotection are accordingly needed prior a claim as a potential therapeutic agent against several oxidative stress-associated liver injuries.

### 6.5. Curcumin and Hepatotoxicity

In terms of drug toxicity, the liver is often the targeted organ. More than 1000 drugs can cause toxicity in the liver and subsequently induce oxidative stress, steatosis and cell death. Most of anti-cancer, anti-analgesic and anti-inflammation drugs and antidepressants can be hepatotoxic. The production of ROS and RNS as the primary events, mitochondrial dysfunction and lipid dysmetabolism as the principal mechanisms of drug toxicity can be mentioned [103,104]. The main problem with these medications is the usage of high doses, which usually lead to hepatotoxicity in humans and experimental animals. Paracetamol (acetaminophen), commonly used as an antipyretic, is one of the most widespread drug-induced liver damage [105]. Manal et al. showed that curcumin supplementation at doses of 50 and 100 mg/kg/day to experimental rabbits with paracetamol-induced hepatotoxicity lowers the elevated aspartate transaminase, alkaline phosphatase and alanine transaminase levels and raises the total protein and albumin levels in plasma. In addition to these changes, curcumin increased the levels of red blood cells and platelets [105]. In another study, the efficacy of curcumin to manipulate the protein content, Succinate dehydrogenase (SDH), TBARS, Adenosine triphosphatase activity (ATPase), alkaline phosphatase activity (ALKase), acid phosphatase activity (ACPase), SOD and body weight of chloroquine phosphate (CQ)-induced hepatotoxicity was observed in a rat model [106]. Propanil (3,4-dichloropropioanilide), used to control weeds in wheat and rice products, is one of the most high-risking herbicide for humans. In a rat model of propanil-induced hepatotoxicity, curcumin improved the effects of propanil intoxication by decreasing lipid peroxidation levels and restored the levels of serum enzymes and reduced glutathione [107]. Although the liver has a high metabolic capacity, it is susceptible from a number of toxins such as thioacetamide (TAA) that causes the rise of ROS levels and the activation of HSCs [104]. Fazal et al. contributed to a study showing the protective effects of curcumin on liver toxicity in a rat TAA model by suppressing oxidative stress and angiotensin-converting enzyme (ACE) gene expression, protecting the liver tissue and anti-oxidant enzymes and restoring hepatocytes [108]. Additionally, Shapiro et al. confirmed the hepatoprotective effect of curcumin by reporting decreased levels of TBARS, minimized oxidative stress and inhibited inducible nitric oxide (iNOS) protein and NF-κB in acute thioacetamide hepatotoxicity rats supplemented with 200 and 400 mg/kg per day curcumin [109]. The increased production of reactive oxygen intermediates and lipid peroxidation, migration of activated PMNs into the liver, severe oxidative stress and eventually extensive damage to the liver are induced by lipopolysaccharides (LPS). Kaur et al. studied the effect of treatment of hepatotoxicity induced by LPS on enzymes and oxidative stress in rat liver. The results showed that the levels of ALT, AST and ALP as well as bilirubin in serum were significantly decreased, while the cytotoxic effects of NO, oxygen free radicals and cytokines were remarkably prevented [110]. Due to the accumulation of nanoparticles in different organs of experimental animals, including liver, spleen, brain and so forth, administration of NZnO (50 mg/kg/day) and treatment with curcumin (200 mg/kg/day) were measured in rats exposed to hepatotoxicity. The increased serum ALT, AST and ALP activity and MDA, decreased GPx and SOD levels suggests a link between NZnO and hepatic oxidative stress [111]. Although sodium fluoride is a substance that induces toxicity and oxidative stress, Moghaddam et al. showed that the protective effect of curcumin was related to its ability to adjust the imbalance of antioxidant enzymes and reduced lipid peroxidation levels in rat with fluoride-induced hepatotoxicity [112]. 60–70% of synthetic dyes are azo dyes that are soluble in processed food. It causes environmental contamination during food processing and can thus have harmful effects on human lymphocytes through direct interaction with genetic material such as DNA [113]. It has been reported that the azo dye tartrazine (Tz) can cause pathological changes in the liver and kidneys. EL-Desoky and colleagues administered doses of 1.0, 2.0, or 4.0 g of curcumin to a rat model of Tz-mediated hepatotoxicity and oxidative stress. They noticed that maximum improving effects on antioxidant enzyme activities were observed when 2.0 g of curcumin was used compared to those exposed to Tz alone [101]. Random or intentional intake of high doses of Cr compounds, such as potassium dichromate (K_2_Cr_2_O_7_), can lead to serious damage to the liver. This damage is generated by increasing lipid peroxidation and ROS levels and inhibiting structural tissue injury, antioxidant enzymes and mitochondrial damage [114,115]. García-Niño et al., in a model of liver oxidative stress induced by K_2_Cr_2_O_7_, remarked that 400 mg/kg curcumin could prevent the increased activities of plasma enzymes, while 15 mg/kg K_2_Cr_2_O_7_ is unable to induce alterations in the oxidative stress markers [115]. Similarly, the protective effect of curcumin (400 mg/kg) was evaluated by García-Niño and colleagues on the hepatotoxicity provoked by K_2_Cr_2_O_7_ (15 mg/kg) in rats. Prevention of histological damage, decrease in body weight gain, increase of liver weight, liver/body ratio and amelioration of the liver oxidative damage can be mentioned as their result [114].

### 6.6. Curcumin and Liver Fibrosis and Cirrhosis

If the development of liver disease is not prevented, it can progress from simple steatosis to more severe disease forms, including hepatitis, fibrosis and cirrhosis [103]. Collagens are the most abundant protein in the extracellular matrix and HSCs are the main cells that produce collagen in the liver. Fibrosis is characterized by an excessive deposition of collagen between hepatocytes and sinusoids [104,116]. Chronic tissue injury, ROS, inflammatory cytokines and apoptotic signals activate the HSC [104,116]. Transforming growth factor beta (TGF-β) also has a critical role in initiating and the development of the HSC phenotypic activity [117]. In addition, oxidative stress increasingly activates HSCs [118]. If the imbalance between synthesis and destruction of collagen by membrane-bound metalloproteinase continues, the fibrosis advances to become cirrhosis. Cirrhosis is the end-stage of progressive fibrosis and is characterized by the degradation of the hepatic lobules structures and blood flow failure [104,117]. CCl_4_ is one of the causative of liver damage, developing fibrosis and further cirrhosis. CCl_4_ initiates cell damage by producing free radicals and increasing collagen synthesis through lipid peroxidation mechanism. Hence, the efficacy of curcumin has been examined in hepatic fibrosis induced by CCl_4_ in rats. It was concluded that treatment with curcumin significantly reduced serum and tissue cholesterol profiles, GOT, GPT, ALP and TBARS [119]. Bruck et al. showed that curcumin inhibited the thioacetamide-induced cirrhosis in rats. They also found a decreasing of oxidative stress, hydroxyproline levels, liver histopathology and spleen weights (*p* < 0.001) [117]. Chenari et al. established that treatment with curcumin in the BDL fibrotic rat model significantly reduced low-density lipoprotein (LDL), total cholesterol (TC) and triglyceride (TG) levels, AMPK, carnitine palmitoyltransferase 1A (CPT-1A), isocitrate dehydrogenase 2 (IDH2) and manganese-dependent superoxide dismutase (MnSOD) gene expressions. In addition, it was observed that the level of HDL and protein/gene expression of the silent mating type information regulation type 2, homolog3 (SIRT3) in response to oxidative stress, by reducing ROS level, significantly increased [118]. Curcumin can also be effective in the treatment of chronic hepatic diseases and reducing levels of ALT, γ-GTP, TGF-β expression can be measured in a rat model of BDL-induced liver cirrhosis. 100 mg/kg of curcumin was also capable of decreasing oxidative stress by alterations of glutathione levels [117]. In another study, the effectiveness of curcumin in the treatment of BDL was shown by reducing liver damage and oxidative stress [120]. There is a work performed on a mouse model of LPS-induced acute liver injury by Zhong and colleagues who studied the hepatoprotective role of curcumin. This effect was then demonstrated through reduction of pro-inflammatory cytokines and oxidative stress, increase of liver antioxidant enzymes and decrease of liver apoptosis by inhibiting phosphoinositide 3-kinase/protein kinase B (PI3K/AKT) signaling pathway [121]. Preclinical studies evaluating the effects of curcumin in the prevention/accelerating of oxidative associated liver diseases are listed in Table 1.

### 6.7. Role of Epigenetic Pathway in Protective Effect of Curcumin against Oxidative Associated Liver Diseases

Epigenetics, reversible alterations in gene expression, regulates the chromatin structure modifications and the initiation of transcription by altering the gene transcription without changing the primary DNA sequence [122]. There is a link between oxidative stress, epigenetics and NAFLD through the mitochondria. Epigenetic mechanisms have a crucial role in the pathophysiology of NAFLD and epigenetic causes of oxidative stress contribute to NAFLD [123]. The most studied epigenetic mechanisms are: (a) DNA methylation; (b) histone modifications; and (c) microRNAs (miRs) [122].

DNA methylation, the first discovery of epigenetic regulation of gene expression, is a biochemical modification (methylation) of cytosine-phosphoguanine (CpG) dinucleotides in promoter regions that is regulated by DNA methyltransferases (DNMTs) [124,125]. Increasing the level of Methyl-CpG binding protein 2 (MECP2) activates HSCs and promotes fibrosis by repressing peroxisome proliferator-activated receptor γ (PPARγ) [124]. Also, epigenetic variations in mitochondrial DNA methylation can cause abnormal gene expression in NAFLD [125]. Among post-translational modifications of histones, acetylation that is usually associated with the activation of gene transcription affects the gene expression in NAFLD [122,125]. MiRs, small noncoding RNAs, regulate post-transcriptional gene expression through degradation or repression of mRNAs. Activation and inactivation of HSCs can be controlled by miRs [124]. In addition, several targets for treatment of NAFLD have been proposed by this mechanism [125]. Hence, among epigenetic machineries, miRs are the most widely investigated in NAFLD [122].

It has been demonstrated that curcumin can reduce the occurrence and/or delay the development of HCC by epigenetic mechanisms such as DNA demethylation and histone deacetylases (HDAC)-inhibitory effect [126]. Furthermore, Curcumin can modulate miRs in liver diseases [127]. A novel mechanism suppressing liver fibrosis by Zheng et al., resulted in the inhibition of cell proliferation and up-regulation of the Phosphatase and tensin homologue deleted on chromosome 10 (PTEN) expression through microRNA-mediated control of DNA methylation after curcumin treatment [128]. Liu et al. showed that the administration of curcumin modulated the growth of human breast cancer cell line MDA-MB-361 and induced the Deleted in Liver Cancer 1 (DLC1) expression. In this study CpG demethylation of many tumor suppressor genes was conducted and inhibition of DNA methyltransferase 1 expression by decreasing the expression of transcription factor Sp1 was observed [129]. In another study, Yuan et.al. investigated the effect of the DNA methylation inhibitor 5-Aza-CdR and curcumin on DNA methylation of the PPAR-α gene in NAFLD pathogenesis. They found that gene expression was regulated through epigenetic modifications, DNA methylation levels were reversed and lipid accumulation was improved [130].

## 7. Nanoformulations of Curcumin in Oxidative Associated Liver Diseases

The first report on the bioavailability of curcumin was in 1978 [131]. The study showed that traces of curcumin could be identified in the plasma of rats receiving 50 mg/kg intravenously. This fact indicated the poor absorption of curcumin. Another study showed that low levels of curcumin could be detected in the plasma after oral administration of high dose, representing the low absorption from the intestine [132]. More experiments have been performed in order to understand the reason behind the poor bioavailability and distribution of curcumin in liver, spleen and kidney or heart [131,132,133,134].

These circumstances opened another area of research with the aim of improving the bioavailability either by encapsulation of curcumin in phosphatidyl-choline (Meriva^®^) [134] or by nanoformulation to increase drug delivery. Phosphatidyl-choline coating increased the detectable level of curcumin and its metabolites in the plasma and the liver [134]. Zein, a corn protein, recently was used to improve the bioavailability of curcumin and liver targeting [135]. Zein has been used in many pharmaceutical products due to its low side effects, slow release of the drug and targeting the liver [136,137]. Algandaby et al. formulated curcumin-zein nanospheres and investigated the efficiency of the product in treating liver fibrosis in CCl_4_-treated mouse model. The study confirmed the increase in liver targeting (3.24 fold) by determination of curcumin level in liver and plasma. Curcumin-zein (cur-zein) form decreased aminotransferases and ALP levels. Additionally, absence of fibrosis and ballooning degeneration and fat accumulation reduction in liver sections were also observed. Therefore, cur-zein improved the curcumin antifibrotic activity in comparison to curcumin alone.

A report by Ahmad et al. 2018 [138] described the formation of nanocomposite of curcumin with chitosan. This material increased the hepatoprotective activity of curcumin. Pretreatment of mice with curcumin-chitosan nanospheres elevated the antioxidant enzymes (SOD, GSH, GST and catalase) and protected the mouse from cadmium toxicity. For improving the bioavailability of curcumin, Singh et al. evaluated the efficacy of curcumin-solid lipid nanoparticles (C-SLNs) on CCl_4_-induced hepatic injury in rats compared to curcumin alone. Microemulsification method was used to prepare C-SLNs with size of 147.6 mm. Using of C-SLNs (12.5 mg/kg) was more effective and reduced oxidative stress, histopathological alterations as well as TNF-α, serum ALT and AST [139].

## 8. Clinical Studies Supporting the Efficacy of Curcumin in Oxidative Associated Liver Diseases

The animal studies confirmed the ability of curcumin in lowering lipogenesis, oxidative stress and increasing insulin sensitivity [140,141]. On the other hand, human clinical studies on the bioavailability and efficacy of curcumin have been mainly performed on patients with cancer or diabetes. The results showed that curcumin is characterized by low systemic bioavailability with rapid metabolism in liver and excretion [142].

Two recent articles that investigated the role of curcumin in prevention and treatment of NAFLD, are listed in Table 2 [143,144]. Panahi et al. performed a randomized controlled study with 87 individuals, 44 patients (20 females and 24 males) were diagnosed with NAFLD and categorized into grades from 1 to 3 according to the liver ultrasonography. Patients group received two doses of curcumin (1000 mg/day) in phytosomal capsules. After 8 weeks, liver aminotransferases and ultrasonography with anthropometric parameters were evaluated in patients and the placebo group. Panahi et al. showed the ability of curcumin phytosomal to reduce the body mass index (−0.99 ± 1.25) *versus* the placebo group (−0.15 ± 1.31). Curcumin-receiving group showed a remarkable decrease in liver enzymes AST, ALT, serum glutamic oxaloacetic transaminase (SGOT) and serum glutamate-pyruvate transaminase (SGPT) levels. Moreover, liver sonography was improved within patients treated with curcumin where the portal vein diameter decreased and vein flow was increased. Curcumin also decreased the NAFLD severity by decreasing the fat content in the liver. The aforementioned results by Panahi et al. confirmed the efficacy of curcumin supplementation for short period in treating NAFLD [144].

Another randomized double-blinded placebo study was carried out to investigate the ability of curcumin in treating NAFLD [143]. This study used a lower dose of curcumin (500 mg/day) in amorphous form and for the same short period (8 weeks). Total 80 subjects participated in the clinical study. Only 40 patients received curcumin daily. Rahmani et al. evaluated the anthropometric measurements, liver aminotransferases, low-density lipoprotein-cholesterol (LDL-C), triglyceride, glucose, cholesterol, high-density lipoprotein-cholesterol (HDL-C) and glycated hemoglobin (HbA1c). After 8 weeks of supplementation with curcumin, the waist circumference, Body mass index (BMI) and weight were significantly reduced. In addition, total cholesterol and LDL-C were decreased while HDL-C was elevated. The other biochemical parameters such as aminotransferases (ALT and AST) were reduced by the end of the trial. In order to confirm the ability and efficacy of curcumin in treating NAFLD, a histological sample of liver should be collected but this is an invasive procedure and holds a risk of morbidity. Liver sonography with biochemical parameters is an excellent alternative technique to show the improvement [145]. In this clinical trial, liver sonography showed that curcumin improved the NAFLD sonography grade to 78.9%, where grade 3 was not detected in the participants, grade 2 was detected in only 13.2% of patients and 15.8% were completely improved.

As discussed previously, curcumin has low bioavailability. However, several clinical trials as well as preclinical studies confirmed the protective and therapeutic effects of curcumin in different oxidative associated diseases including liver disorders. Different evidences have suggested that the therapeutic activities of this natural molecule, despite its undesirable pharmacokinetic properties, is mainly due to the main metabolites of curcumin, which may have a key role in the biological function [146,147].

## 9. Conclusions

Curcumin is able to protect and treat liver diseases and to alter different cellular pathways. For instance, curcumin induce the expression of heme oxygenase-1 [148] which cleaves heme and produces CO, biliverdien and bilirubin and other antioxidant molecules [149]. Regarding the reported effects on cellular responses, curcumin inhibits activation and proliferation of HSC, leading to a decrease in production of extracellular matrix collagen and protecting liver from fibrogenesis [83]. This effect on HSC was found to be through the down-regulation of PDGF-βR, EGFR and TGF-β coupled with the reduction of mRNA level of PPAR-γ [150,151]. Curcumin induced the synthesis of reduced glutathione [152] leading to a marked decrease in lipid peroxidation products such as lipid hydroperoxide and MDA [83,84]. The increase in GSH level is due to the ability of curcumin in elevating the gene expression of the rate-limiting enzyme in glutathione synthesis and glutamate cysteine ligase (GCL) [83]. Curcumin treatment leads to a marked decrease in the level of proinflammatory cytokines such as TNF-α, INF-γ, IL-1β and IL-6 [84,153]. The decrease in proinflammatory cytokines was due to the inhibition of NF-κB translocation to the nucleus and decreasing its DNA binding activity [84].

Curcumin increased the expression of SIRT3, a NAD^+^-dependent deacetylase and ADP-ribosyltransferase. SIRT3 activation by curcumin led to the decrease in lipid deposition through AMPK and the mitochondrial CPT-1A. Moreover, SIRT3 caused a decrease in ROS level by upregulating the expression of MnSOD and the mitochondrial IDH2 [118]. Another signaling cellular pathway attenuated in the liver by curcumin treatment is PI3K/Akt. Hence, curcumin inhibited and decreased the level of apoptotic markers such as Bad, Bcl-xL, cytochrome *c*, Apaf-1, cleaved caspase-9, -3 and -6. p38 mitogen-activated protein kinase/JUN was also downregulated and protected the liver cells from death [121]. Also, curcumin downregulated the expression of ACE [108].

A wide variety of preclinical studies support the effectiveness of dietary curcumin in the management of oxidative associated liver diseases. However, there are few RCTs assessing the efficacy of curcumin in liver disorders. Table 2 exhibits the evidence of curcumin in the prevention and treatment of oxidative associated liver diseases in humans. Further well-designed RCTs are therefore required to confirm the dietary and adjunctive role of curcumin as promising protective or curative agent in the management of oxidative associated liver diseases.

To conclude, the results obtained from the present review revealed that curcumin can be effective in various types of oxidative associated liver disorders. This potentiality attributes to curcumin effects on hepatotoxicity, non-alcoholic steatohepatitis, alcoholic liver disease, liver fibrosis and cirrhosis as well as hepatic injury. Experimental evidences indicate that curcumin exhibits its preventive and curative effect against oxidative associated liver diseases through various cellular signaling pathways. Those pathways include ERK/p38/MAPK pathway, hepatic Nrf2/ARE/Keap1 signaling, up-regulation of detoxifying genes expression, TIMP signaling, AMPK pathway and lipid metabolism, as well as down-regulation of Rac1, NOX1 and Rac1-GTP transduction. Regarding the above mentioned biological activities of curcumin in either protecting or treating liver, it is highly recommended to consider curcumin as a safe and effective natural product for oxidative associated liver diseases. Among the studies that were conducted in various oxidative models associated liver disease, some investigated the protective effects of curcumin [83,84,85,86,97,98,105,106,107]. According to these articles, curcumin as a dietary supplement has a protective role against the onset of liver diseases. The intake of a significant content of curcumin in the daily regimen or as dietary supplementation along with restricted therapeutic options can provide perfect prevention and treatment for liver disorders. Present review revealed that further in vitro and preclinical studies are encouraged to recognize the exact bioavailability, bioefficacy and cellular transduction signaling pathways of curcumin in managing oxidative associated liver diseases.

## Figures and Tables

**Table 1:** Preclinical studies evaluating the effects of curcumin in the prevention/accelerating of oxidative associated liver diseases.

Liver Disease Type	Experimental Model Used (Animal, Strain, Genetic or Dietary Liver Injury)	Curcumin Source	Dose and Formulation (Injection)	Duration of Treatment	Reference
**Non-Alcoholic Steatohepatitis (NASH)**					
Ccl_4_ 0.5 mL/kg/every other day/SC/3 weeks	Male Albino Wistar rat	Sigma, St. Louis, MO, USA	200 mg/kg/day in olive oil (oral)	3 weeks	[58]
Methionine and choline deficient diet (MCD diet)	Male C57BL/6 mice	Sigma, St. Louis, MO, USA	25 µg/every other day/in DMSO (IP)	4, 8 or 10 days	[59]
High fat diet (HFD)	Adult Sprague-Dawley rats	Sigma, St. Louis, MO, USA	50 mg/kg/day/suspended in 0.5% CMC	6 weeks	[60]
200 μg STZ/single dose/SC/2 days after birth (HFD 32)	C57BL/6 J mice	Sigma-Aldrich, Tokyo, Japan	100 mg/kg/day in 1% gum Arabic (oral)	14 weeks	[61]
**Alcoholic Liver Disease**					
50% ethanol (7.5 g/kg/day/4 weeks/oral)	Female Sprague-Dawley rats	Cayman Chemical Company, USA	400 or 600 mg/kg/twice a day/in 50% ethanol	4 weeks	[63]
25% ethanol (5 g/kg/day/6 weeks/oral)	Male ICR mice	Sigma, St. Louis, MO, USA	19.7 or 47.5 mg/kg/day (oral)	6 weeks	[64]
100 mm ethanol for 8 h	Primary rat hepatocytes from male Sprague–Dawley rats	diferuloylmethane; CAS No. 458-37-7, Sigma, St. Louis, MO, USA	5–50 M/dissolved in 0.5 N NaOH then diluted in PBS	0–5 h before ethanol treatment	[65]
2.4 g/kg/day ethanol for the initial 4 weeks and 4 g/kg/day for another 2 weeks	Male Balb/c mice	Diferuloylmethane; CAS No. 458-37-7), Sigma, St. Louis, MO, USA	75 mg/kg/day/in DMSO (oral)	6 weeks along with ethanol intake	[66]
2.4 g/kg/day ethanol/daily/6 weeks	Adult Male Balb/C mice	Purity >98.0%, from the National Institute for Food and Drug Control (Beijing, China)	75 or 150 mg/kg/in olive oil/(oral)	1 h before ethanol administration for 6	[67]
Low *ω*-3 poly unsaturated fatty acids (PUFA) diet + ethanol High 𝜔 − 3 PUFA + ethanol	Female Wistar-Furth rats	-	150 mg/kg/day	8 weeks	[68]
5% ethanol/IV, 5 times a week/100 µL/mouse/for 2 weeks	Male C57BL/6 mice	Sigma-Aldrich, USA	1 mM or 1 mM/100 µL/mouse (IV) dissolved in 0.5 N NaOH then diluted in PBS	5 times a week for 2 weeks	[69]
**Oxidative Stress Inducers**					
H_2_O_2_ 0.5% (*v*/*v*)/day/for 60 days	Male Wistar rats	Crude curcumin was purchased from local wet market in Baghdad, Iraq	200 mg/kg/day	1st model = 30 days after induction of oxidative stress 2nd model = 15 days then followed by receiving H_2_O_2_ for 60 days	[70]
Methotrexate 20 mg/kg/I.P./single dose	Rat		100 mg/kg/day (I.P.)	5 days	[72]
Melathion (MAL) 200 mg/kg/oral	Female Sprague Dawley rats	*Curcuma longa* Turmeric, Sigma, St. Louis, MO, USA (C1386)	1 g/kg (oral)	1 days	[76]
Iron overload (Haemojet^®^) containing ferric hydroxide polymaltose 100 mg/Kg/I.P./3 doses per week/for 2 weeks	Male albino rats	Purity~95%, Indian production, purchased from El-Goumhoria Co., Cairo-Egypt	100 mg/kg/day/dissolved in DMSO	3, 4 or 5 weeks	[75]
2,3,7,8-tetrachlorodibenzo-p-dioxin 2 mg/kg/week/oral diluted in corn oil	Female Sprague Dawley rats	Sigma Chemical Co., St. Louis, Missouri, USA	100 mg/kg/day/dissolved in corn oil	30 and 60 days	[77]
Turpentine oil 0.6 mL/kg/I.M.	Wistar Bratislava albino rats	Purity >98%, Abcam (Cambridge, United Kingdom)	150 mg/kg/dissolved in 0.5% CMC (oral)	1st model = 60 min prior Turpentine injection 2nd model = 120 min after Turpentine injection	[78]
Cdcl_2_ 0.025 mmol/kg to rats and 0.03 mmol/kg to mice/S.C.	Adult male Wistar rats and male CD mice	Sigma–Aldrich, St. Louis, MO, USA	50 mg/kg/day/dispersed in 0.25% methylcellulose	3 days	[81]
Immobilization-induced stress, rats kept in the restrainers for 1 h every day, for 21 consecutive days	Wistar albino rats	Sigma-Aldrich Chemical (St. Louis, USA)	10 or 20 or 30 mg/kg/day/IP	21 days	[82]
Ccl_4_ 1 mL/kg (1:1) in olive oil/every other day for 8 weeks./I.P.	Male Sprague-Dawley rats	-	200 or 400 mg/kg/suspended in PBS	48 h	[83,84]
Lindane 1st model = 60 mg/kg/for 24 h/oral 2nd model = 30 mg/kg/for 14 d	Male Wistar rats	Sigma Aldrich (St. Louis, MO, USA)	100 or 200 mg/kg/day/dissolved in DMSO (oral)	1st model = pretreatment for 14 days 2nd model = posttreatment for 14 days	[85]
Cypermethrin 25 mg/kg/day/for 28 days	Adult male Wistar rats	Sigma Chemicals, USA and SRL Chemicals, India.	100 mg/kg (oral)	28 days	[86]
Trichloroethylene (TCE) 1.2 mmol/kg/diluted in corn oil/24 h	Male ddY mice	-	10, 50 or 100 µM/dissolved in DMSO (I.P.)	24 h	[87]
**In Vitro Study**					
Quinocetone (QCT)-	Human hepatocyte L02 cells	Purity 98%, Aladdin Reagent Co., Ltd. (Shanghai, China)	2.5 or 5 mM/0.1% DMSO	2 h pretreatment then incubated for 4 or 24 h with QCT	[79]
Glucose oxidase (GO) 100 mu/mL/2 h	Rat HSCs-6	Sigma-Aldrich (St. Louis, MO, USA)	0.15 µM	3 h pretreatment then incubated with GO for 2 h	[80]
**Liver Injury**					
LPS (10 μg/kg/I.P.)/D-galactosamine (400 mg/kg/I.P.)/24 h	Male Wistar rats	Sigma-Aldrich (Prague, Czech Republic)	100 mg/kg (I.P.)	Pretreatment for 1 h	[88]
Microcystins 38.11 μg/kg/3 h/I.P.	Male Swiss mice		300 mg/kg (oral)	7 days pretreatment	[89]
Biliary duct ligation (BDL)	Male Wistar albino rats (*Rattus novegiccus*)	curcumin (97%, purity) from Sigma Chemicals	50 mg/kg/day in corn oil (oral)	14 days	[90]
	Male Wistar rats	Curcumin (purity >80%) from Sigma Chemicals	100 mg/kg/day in Carboxymethyl cellulose (CMC) (oral)	28 days	[91]
Ischemia/reperfusion (I/R)	Female Wistar Albino rat	Sigma Chemical Co., USA	100 mg/kg (I.P.)	30 min pretreatment before I/R	[92]
Acetaminophen (APAP) (750 mg/kg/single dose/oral	Male Albino Wistar rats	Armal company	200 mg/kg in corn oil (oral)	1st = 24 h before APA 2nd = 2 h after APAP 3rd = 12 h after APAP	[94]
Gentamicin (100 mg/kg/I.P.)	Male albino rats	Sigma Chemical Co.	20 mg/kg/every other day in 1% CMC (oral)	21 days	[95]
**Xenobiotics**					
Aflatoxin B1 (25 μg/kg)	Male Fischer rats	Sigma Chemical Company	200 mg/kg	90 days	[96]
Lambda cyhalothrin	Male albino rats (*Rattus norvegicus*)	Sigma-Aldrich Chemical Co	200 mg/kg/day suspended in PBS (oral)	4 weeks	[97]
Hg (0.6, 1.2, or 2.4 mg/kg in saline/IP/daily/3 days)	Male and female Adult Wistar rat	Curcumin (98%) was provided by Sigma, Saint Louis, Missouri, USA	100 mg/kg/in DMSO (SC)	2 h pretreatment before Hg	[100]
**CCl_4_**					
(1:1 in olive oil) 1 mL/kg/every other day/IP/4 weeks	Male Sprague-Dawley rats	*Curcuma longa* L. (CLL, turmeric)	200 mg/kg/day in PBS (oral)	4 weeks	[98]
1st model = 0.2 mL/kg/24 h 2nd model = 1 mL/kg (1:1 in corn oil)/2 times per week, oral/4 weeks	Male Sprague-Dawley rats	*Curcuma longa* L. (*Zingiberaceae*)	50, 100 or 200 mg kg/day in corn oil (oral) orally for 4 consecutive days	1st = 4 days before CCl_4_ treatment 2nd = during CCl_4_ treatment for 4 weeks	[99]
30% CCl4 in olive oil (0.05 mL/10 g/IP	Fish *Cyprinus carpio* var. Jian (Jian carp)	Sigma-Aldrich Chemical Co	0.1%, 0.5%, or 1.0%	60 days before CCl_4_ treatment	[102]
**Hepatotoxicity**					
Propanil 20 mg/kg/3 times a week/in olive oil (oral)	Albino rat	Sigma Chemical, USA	50 mg/kg/3 times a week/in olive oil	28 days	[95]
Paracetamol 500 mg/kg/day for 15 days (oral)	Adult male rabbits	Sigma Chemical	50 and 100 mg/kg/in corn oil (oral)	15 days	[105]
Chloroquine phosphate (CQ) 100, 200 or 300/daily/45 d	Male Swiss Albino mice	-	80 mg/kg/day (oral)	45 days during CQ treatment	[107]
TAA 200 mg/kg/I.P. for 12 weeks	Male Wistar albino rats	-	75 mg/kg (oral)	12 weeks after discontinuation of TAA	[108]
TAA 300 mg/kg/2 days/I.P./dissolved in a solution of glycerol formal, chremaphore and H_2_O (5:2:2)	Male Wistar rats	Sigma Chemical	200 or 400 mg/kg/day dissolved in glycerol formal, chremaphore and H_2_O (5:2:2) (oral)	48 h before TAA administration then continued during the two days of TAA injection	[109]
LPS 1 mg/kg/I.P.	Male Wistar rats	Sigma Aldrich Chemicals Private Ltd., New Delhi, India	5, 30 or 60 mg/kg/suspended in 0·5% CMC (oral)	6 days before LPS injection and sacrificed after 6 h post LPS injection	[110]
Nzno 50 mg/kg/on 7th day of saline administration (oral)	Male Wistar rats		200 mg/kg/day/in corn oil (oral)	7 days prior NZnO and continued for 21 days	[111]
Naf 600 ppm via drinking water/7 days	Male Wistar rats	Sigma-Aldrich Chemical, USA	10 or 20 mg/kg/dissolved in 5% DMSO (I.P.)	7 days then exposed for 7 days NaF	[112]
(Tz, azo dye) 7.5 mg/kg/diet/90 days	Male Wistar Albino rats	Local markets, Saudi Arabia	1, 2 or 4 g/kg	90 days	[113]
K_2_Cr_2_O_7_ 15 mg/kg/I.P./single dose	Male Wistar rats	Sigma-Aldrich (St. Louis, MO, USA).	400 mg/kg/suspended in 0.5% CMC (oral)	10 days prior single dose of K_2_Cr_2_O_7_ for 24 h or 48 h	[114,115]
**Fibrosis and Cirrhosis**					
TAA 200 mg/kg/I.P./twice a week for 12 weeks	Male Wistar rats	-	300 mg/kg/day/in solvent/2 mL per rat (intragingival)	12 weeks along with TAA or 4 or 6 weeks after TAA discontinuation	[116]
Biliary duct ligation	Male Wistar Albino rats	Sigma-Aldrich, USA	100 mg/kg/day (oral)	1st dose = 3 days before BDL and terminated after 14 days	[120]
	Male Wistar Albino rats	Sigma Chemicals Co Purity (HPLC) >80%, USA	100 mg/kg/day/suspended in 5% CMC (oral)	28 days after BDL surgery	[118]
	Male Wistar rats	Sigma Chemicals Co, USA	100 mg/kg/day/suspended in 0.7% CMC (oral)	28 days	[117]
CCl_4_ 0.4 g/kg/3 times per week/dissolved in mineral oil/for 3 months	Male Wistar rats	Sigma Chemicals Co, USA	100 mg/kg/day (oral)	2 months	[117]
LPS 5 mg/kg/I.P.	Male C57BL/6 mice	-	20, 40 or 80 mg/kg/day (oral	4 weeks	[121]

**Table 2:** Clinical studies of curcumin used in the treatment of oxidative associated liver diseases.

Dose	Study Design	No. of Patients	Duration of Treatment	Result	Reference
1000 mg/day	Patients with NAFLD were randomly assigned to the curcumin (*n* = 44) or placebo group (*n* = 43)	87	8 weeks	↓ the body mass index, AST, ALT, SGOT, SGPT	[144]
500 mg/day	Patients with NAFLD were randomly assigned to the curcumin (*n* = 40) or placebo group (*n* = 40)	80	8 weeks	↓ Total cholesterol, LDL-C, ALT, AST ↑ HDL-C	[143]
